# Supporting Looked After Children and Care Leavers In Decreasing Drugs, and alcohol (SOLID): protocol for a pilot feasibility randomised controlled trial of interventions to decrease risky substance use (drugs and alcohol) and improve mental health of looked after children and care leavers aged 12–20 years

**DOI:** 10.1186/s40814-017-0138-7

**Published:** 2017-05-22

**Authors:** Hayley Alderson, Ruth McGovern, Rebecca Brown, Denise Howel, Frauke Becker, Louise Carr, Alex Copello, Tony Fouweather, Eileen Kaner, Paul McArdle, Elaine McColl, Janet Shucksmith, Alison Steele, Luke Vale, Raghu Lingam

**Affiliations:** 1 Institute of Health and Society, Newcastle University, Baddiley Clarke Building, Richardson Road, Newcastle, NE2 4AX UK; 2 School of Psychology, University of Birmingham, Edgbaston, Birmingham, B15 2TT UK; 3 Child and Adolescent Mental Health Services, Northumberland, Tyne and Wear NHS Foundation Trust, St Nicholas Hospital, Jubilee Road, Gosforth, Newcastle, NE3 3XT UK; 4 Health and Social Care Institute, School of Health & Social Care, Teesside University, Middlesbrough, TS1 3BA UK; 5 Newcastle upon Tyne Hospitals NHS Foundation Trust, Freeman Hospital, Freeman Road, High Heaton, Newcastle Upon Tyne, NE7 7DN UK

**Keywords:** Looked after children, Drugs and alcohol, Feasibility, Social work, Intervention

## Abstract

**Background:**

Looked after children (LAC) and care leavers are young people who have been placed under the legal care of local authorities, in many instances due to a history of abuse and/or neglect. These young people have a significantly increased risk of substance use and mental disorder compared to their peers. The aim of the SOLID study is to assess the feasibility and acceptability of a definitive three-arm multi-centre randomised controlled trial (RCT) that compares the effectiveness of two interventions that aim to reduce risky drug and alcohol use and improve mental health among LAC aged 12 to 20 years with usual care.

**Methods:**

All LAC aged 12 to 20 years residing in four local authorities in North East England will be screened by their social worker for risky drug and alcohol use using the CRAFFT (Car, Relax, Alone, Forget, Friends and Trouble) screening tool. Those who score ≥2 will be invited to take part in the trial after further eligibility checks. Informed consent will be taken and baseline data collected. Participants will then be randomised into either (i) Motivational Enhancement Therapy, (ii) Social Behaviour and Network Therapy, or (iii) control–usual care. Follow-up data will be collected 12 months post-baseline. The baseline and follow-up questionnaires will measure self-reported drug and alcohol use, mental health and well-being and health-related quality of life. The follow-up will also collect data on placement stability and self-reported sexual, antisocial and criminal behaviour. Participants will also be asked about the use of health and social services. A detailed process evaluation, using both qualitative and quantitative methods, will be conducted and involve LAC, their carers, social workers and drug and alcohol practitioners.

**Discussion:**

Despite having an increased likelihood of risky substance misuse, there is a lack of evidence outlining specific interventions to decrease drug and alcohol use targeting LAC. This feasibility study will provide the information needed to develop a definitive trial. LAC will benefit from the results of this study and the further development of the interventions.

**Trial registration:**

ISRCTN80786829

## Background

Drug and alcohol (substance) use in young people is a major public health problem, which causes a significant economic strain on healthcare and society [1]. In high-income countries, substance use accounts for 11% of the total burden of disease, calculated as disability-adjusted life years lost [2]. It was estimated in 2014 that alcohol-related harm costs the UK £21 billion annually, with an additional £15.4 billion estimated to result from drug addiction [3]. Research from the USA, UK and Sweden demonstrates that risky substance use in adolescence predicts adult alcohol and drug use and significantly increases the risk of adult mental disorder, crime and poverty [4–6]. A comparison of substance use rates among 15- and 16-year-olds across 36 European countries found that although there has been an overall fall in drug use in teenagers over the last decade, the UK is in the top five countries for lifetime use of cannabis and other illicit drugs and in the top ten with regard to binge drinking (heavy sessional or risky single-occasion drinking) [7]. Furthermore, there has been a rapid increase in the use of novel psychoactive substances (NPS, previously known as ‘legal highs’) in the UK, Europe, Australia and USA, which are likely to have significant physical and mental health consequences [8–10].

In the UK context, looked after children (LAC) are children up to the age of 18 who are under the legal care of local authorities [11]. Such young people are described as being ‘out-of-home’ care in both USA and Australia [12, 13]. Care leavers are young adults who are no longer legally looked after, but who are entitled to support from their local authority. Care leavers are typically aged 18 to 21 but can range from 16 to 25 depending on their circumstances, such as being in education [14, 15].

A total of 70,440 children and young people were ‘looked after’ by local authority services (i.e. local government) in England for the year ending 31 March 2016, an increase of 1% compared to 31 March 2015 and an increase of 5% compared to 2012 [16]. This year, the number has increased partially due to the 1470 unaccompanied asylum-seeking children that have arrived in the UK [16].

The 2014 National Institute for Health and Care Excellence (NICE) guideline, ‘Interventions to reduce substance misuse among vulnerable young people’, in the UK, highlighted LAC as a group vulnerable to substance use [17]. About 7% of the approximately 21,000 young people accessing specialist drug and alcohol services in the UK in 2012 self-reported that they were in care [18]. LAC have multiple risk factors for substance use, poor mental health, school failure and early parenthood [19]. These factors include parental poverty, absence of support networks, parental substance use, poor maternal mental health, early family disruption and, in the majority of cases, abuse and/or neglect [20, 21]. LAC, aged 11 to 19 years, have a fourfold increased risk of drug and alcohol use compared to children not in care [22]. Twenty-five percent of LAC aged 11 to 19 years and 42% of young people in residential care drank alcohol at least once a month, compared to 9% of young people not looked after [22]. A national survey of care leavers showed that 32% smoked marijuana daily, and data from 2012 showed 11.3% of LAC aged 16 to 19 years had a diagnosed substance use problem [23, 24]. In addition, research from Britain found that LAC have a nearly fivefold increased odds of at least one mental health diagnosis than their non-LAC peers (disorders including anxiety, depression or behavioural disorders) (OR 4.92; 95% CI 4.13, 5.85), further increasing their risk of substance use and poor life chances [25].

The long-term outcomes for LAC in terms of health, education, employment and risk of criminality is poor, resulting in a significant cost to society and increased risk of intergenerational poverty. Forty percent of 20-year-olds who have been in the care system in the UK are not in education, employment or training (NEET) [24]. Data from the 1970 British birth cohort at age 30 years showed that men with a history of being in local authority care were twice as likely to be unemployed (OR 2.6; 95% CI 1.4, 5.0), have a criminal conviction (OR 2.3; 95% CI 1.5, 3.4) and have been seen for a mental health-, drug- or alcohol-related problem after the age of 16 years (OR 1.7; 95% CI 1.1, 2.6) than non-looked after peers [26]. There are limited longitudinal data looking at the impact of drug and alcohol use on young people as they move into adulthood. However, research from the criminal justice system in Scotland showed that 34% of youth offenders had been in care. Of these offenders, 75% reported drug use (compared to 57% of those not previously in care) [27]. Research from the USA and Australia similarly shows that young people within the care system tend to have poorer outcomes, such as being more likely to be unemployed and to have mental health difficulties, than young people who grew up in their biological home [12, 13].

Effective interventions in LAC could have a beneficial effect on the long-term mental and physical health of these vulnerable young people, reduce health inequality and, due to their increased risk of early parenthood, potentially impact intergenerational health. Unfortunately, there is limited research, including cost-effectiveness data and, at present, no national guidelines on the most effective interventions to decrease risky drug and alcohol use in this group. This lack of data was highlighted by the Chief Medical Officer (CMO) annual report 2012, which stated that one of the key research areas was to assess the most effective interventions to reduce multiple risk-taking behaviours, including drug and alcohol use in this group [28].

Two existing interventions have shown to be effective in decreasing substance use: Motivational Enhancement Therapy (MET) and Social Behaviour and Network Therapy (SBNT). MET is a client-centred, directive counselling approach developed as a concentrated version of motivational interviewing which adds a problem feedback component to standard treatment [29, 30]. The intervention has been shown to decrease substance use in a range of participants including adolescents [30, 31]. The basic assumption of MET is that the motivation and responsibility for change lie within the client, and it is the therapist’s role to create an environment to enable the client to change. Unlike other approaches, ambivalence is assumed to be the norm and motivation is formed and enhanced within the context of the therapist-client relationship. The therapist employs specific strategies to build and strengthen motivation by eliciting self-motivational statements from the client.

SBNT is a systematic counselling approach, which utilises cognitive and behavioural strategies to help clients build social networks supportive of positive behaviour change in relation to problem substance use and goal attainment [32]. SBNT is based on the premise that social network support for change is key in helping people deal effectively with addictive behaviour. The intervention focuses on addressing substance use by engaging with a network of positive support for lifestyle change. This work is conducted in collaboration with the young person with whom early identification of the social network is carried out. An important aspect of SBNT, especially important for LAC, is that it aims to sustain engagement with vulnerable young people by widening the reach of the intervention beyond the traditional family to include supportive peers or other figures perceived as being important by the young person, e.g. teachers, social workers or possibly wider family members such as grandparents.

The formative part of the SOLID study adapted SBNT and MET, for use with LAC. The adaptations were based on qualitative research with LAC, carers, social workers and drug and alcohol practitioners. Formative findings and a discussion of the intervention development process will be published separately.

### Aim

The SOLID pilot feasibility trial (Supporting Looked After Children and Care Leavers In Decreasing Drugs, and alcohol) aims to investigate whether it is possible to recruit and retain, to 12-month follow-up, LAC aged 12–20 years, who screen positive for drug and/or alcohol use, into a randomised controlled trial of behaviour change interventions to reduce risky substance use (illicit drugs and alcohol) and improve mental health.

The specific objectives of the pilot feasibility trial are:

- To establish response rates, variability of scores, data quality and acceptability of the proposed outcome measures for self-reported alcohol and drug use, health-related quality of life, mental health and well-being, sexual behaviour and placement stability 12 months post-baseline, in order to design a definitive multi-centre RCT
- To assess engagement and participation with the adapted Motivational Enhancement Therapy and Social Behaviour and Network Therapy based interventions by LAC, their support network and frontline drug and alcohol workers
- To assess the fidelity of intervention delivery by drug and alcohol workers and pilot a tool for measuring fidelity of the adapted interventions
- To develop cost assessment tools, assess intervention delivery costs and carry out a value of information analysis to inform a definitive study
- To apply pre-specified ‘stop/go criteria’ and determine if a definitive multi-centre randomised controlled trial is feasible and, if so, develop a full trial protocol

Clear success (‘go’) criteria for this feasibility trial would be for at least 60% of eligible participants to consent to taking part in the trial, to successfully recruit 50 LAC per arm and retain at least 70% of participants at 12-month follow-up. In addition, data from interviews and focus groups should indicate whether trial processes are acceptable to staff and LAC. The individual interventions will be assessed as feasible if the intervention can be delivered with fidelity and at least 80% of the participants attend 60% of the intervention sessions.

## Methods/design

**Fig. 1:**
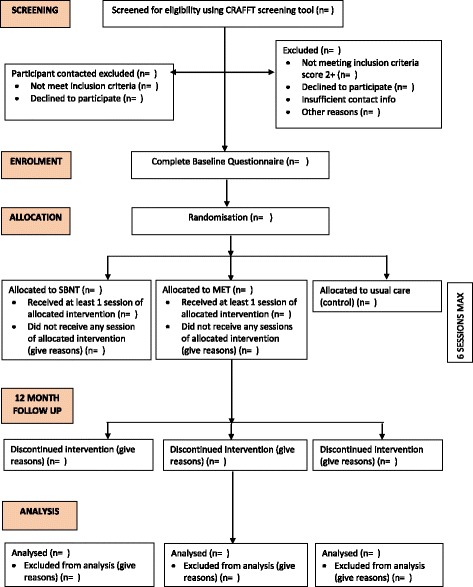
SOLID study CONSORT diagram. *SBNT* Social Behaviour and Network Therapy, *MET* Motivational Enhancement Therapy

The three-arm RCT compares MET and SBNT to usual care and involves LAC and care leavers across four local authorities in North East England. Trial participants will be recruited via a screening measure administered by social workers. Data will be collected at baseline and 12 months post-baseline. Participants will be randomised after completing the baseline questionnaire. Those allocated to the control group will receive usual care, which involves their social worker making a referral along the usual drug and alcohol service pathway as required. Figure 1 presents the study flow diagram and will identify the number of young people screened, enrolled into the study, allocated to each intervention arm and contacted at 12-month follow-up.

### Setting

The study is taking place across six local authority areas in North East England (Newcastle, Durham, Redcar and Cleveland, Middlesbrough, Gateshead and Stockton). 

### Recruitment and screening

Participants will be screened via social work teams in each local authority. All LAC aged 12–20 years in the study sites will be screened using the validated six-question CRAFFT (Car, Relax, Alone, Forget, Friends and Trouble) tool [33–35]. A score of 2 or more indicates risky substance use. The CRAFFT has been used extensively with young people and is sensitive and specific in identifying problem substance use [33–35]. Social workers will screen LAC during routine appointments and provide them with a brief initial contact leaflet. This will capture the young person’s contact details and ask if they consent to being contacted to receive more information about the study.

Those who score ≥2 on the CRAFFT tool and who are able to provide informed consent in English are eligible to participate in the trial. Those who score <2, who are currently receiving treatment from drug and alcohol services, are due to move out of the area or are unable to give informed consent (due to acute or severe mental health difficulties, mental capacity or language barriers), are ineligible to participate.

The research team will contact eligible participants who have expressed an interest in the study.

### Consent to participate

The young person must first agree to being contacted by a researcher to learn more about the study. Those who are eligible and consent to be contacted will be telephoned by a researcher who will check inclusion and exclusion criteria, provide more information about the study and invite them to participate. If the young person consents to participating in the trial, the researcher will arrange to visit the participant to obtain written consent and administer the baseline questionnaire.

For those aged 16 years and over, informed consent will be taken directly. Information on the study will be shared with parents/carers as appropriate. For participants aged under 16, consent must also be obtained from an adult with parental responsibility (PR). Those aged 12 to 15 must be seen by the researcher with an accompanying adult (such as a parent, carer, social worker or children’s home lead) who will be asked to provide informed consent on behalf of the young person. If the accompanying adult does not have PR, the research team will contact the adult with PR to obtain informed consent. If the parent is not contactable, or it is deemed by the designated social worker that it would pose a risk to the young person for their parent to be contacted, the social worker/local authority guardian with PR will be contacted to sign the consent form.

### Randomisation and blinding

Once the participant has completed the baseline questionnaire, they will be allocated to a trial arm by the project secretary. Individual randomisation to the three trial arms will be stratified by placement type (residential/non-residential), site and age band (12–14/over 14) to reflect risk profile for substance use. Randomisation will be administered centrally via Newcastle Clinical Trials Unit using a secure web-based system.

Blinding of group allocation will not be possible for the LAC who will complete the self-report follow-up assessment or for those delivering the intervention. The trial statistician and health economist will only be unblinded after the final analysis or if requested to do so due to safety concerns expressed by the Trial Oversight Committee.

### Sample size

As this is a feasibility study, a formal power analysis for the sample size is inappropriate. The aim is to estimate the standard deviation for a continuous primary outcome (number of occasions drinking 5+ standard drink units in a single occasion as derived from the TLFB/30 at 12-month follow-up) to the necessary degree of precision, so that a power analysis for a future definitive trial can be carried out. It has been shown that data from a minimum of 35 respondents in each trial arm will provide the necessary precision for a continuous outcome [36]. Assuming a 30% loss to follow-up, the sample size to be recruited will be inflated to 50 young people in each of the three arms.

### Interventions

#### Control

LAC within the control arm will receive usual care via their social worker with a referral along usual drug and alcohol service pathways as required.

### Motivational Enhancement Therapy

Whilst the adapted intervention retains the essential components of MET described above, the formative research findings have resulted in the intervention being extended from three 50-min sessions to a maximum of six sessions, each of up to 1 h duration. The initial session focuses upon developing a trusting relationship between the LAC and the practitioner; session 2 is concerned with the LAC discussing the nature of their substance use; session 3 consists of an individual cost-benefit analysis of the LAC’s substance use; session 4 is an individual cost-benefit analysis of change; session 5 focuses on trying to elicit a commitment to change; and session 6 reviews the LAC’s progress in respect of agreed change and commitment to sustain change. However, as the intervention is delivered to meet the young person’s needs, it could be completed in less than six sessions if appropriate. Further, drug and alcohol practitioners are encouraged to consider a range of approaches to elicit self-motivational statements from the LAC in addition to traditional talking approaches. This includes creative writing, arts and crafts and the completion of worksheets.

### Social Behaviour and Network Therapy

The adapted SBNT adheres to the essential components of the intervention described above; however, the formative research has resulted in the intervention being decreased from eight 50-min sessions to six sessions lasting up to 1 h. The initial session focuses on conducting a review of the young person’s social network. Sessions 2–5 consist of delivering a combination of core topics to build positive support for change. Topic 1 focuses on deciding goals, eliciting commitment, agreeing to the plan and recruiting the network. Topic 2 focuses on communication and coping, and topic 3 focuses on lifestyle changes and increasing pleasant activities. Session 6 is the final session and consists of a review of progress made, planning for the future and ending the treatment intervention. However, as MET is delivered to meet the participant’s needs, it could be completed in less than six sessions if appropriate. As identified with the MET approach above, practitioners are encouraged to consider a range of approaches to engage the LAC and their support network. The approaches can include a mixture of the traditional therapeutic approach, creative sessions and more formal worksheets.

### Delivery and training

In each of the participating local authority areas, young people’s drug and alcohol treatment is provided by voluntary sector organisations with charitable status. In each area, a drug and alcohol service has agreed to take part in the study by delivering the three treatment arms. The young person will be contacted by the drug and alcohol service for treatment to the appropriate treatment group (MET or SBNT) within 6 weeks of randomisation.

#### The drug and alcohol practitioners

The three interventions (MET, SBNT and control) will be delivered by experienced young people’s drug and alcohol practitioners. The practitioners have varying professional backgrounds including youth work, social work, counselling and unqualified workers. They are experienced in the delivery of drug and alcohol psychosocial interventions for young people. However, none of the practitioners have been trained in MET or SBNT previously. To avoid contamination in each geographical location, teams will be split to ensure different practitioners within each service deliver either MET, SBNT or usual care.

#### Training and supervision

Drug and alcohol practitioners will receive two full days’ training in the adapted allocated intervention, either SBNT or MET. Training for each intervention will take place at a specialist addiction service, and training will be facilitated by two experienced members of the research team. All drug and alcohol practitioners will attend monthly individual supervision sessions; audio recordings of their sessions will form the content of their supervision. In addition, practitioners will be invited to attend two group supervisions with the research team to discuss issues relating to fidelity of intervention delivery. To prevent contamination, group supervisions will be organised separately for practitioners delivering MET and those delivering SBNT. In addition to the planned sessions, practitioners allocated to MET and SBNT will have access to the research team who can also provide support and guidance. No additional supervision will be provided to practitioners within the control arm.

### Measures (baseline and follow-up)

#### Baseline

After informed consent has been obtained but prior to randomisation, the researcher will collect baseline information from the LAC using a self-completed questionnaire, administered via tablet computer. The researcher will be available to answer questions of clarification if needed. This questionnaire will record demographics, placement type, drug and alcohol usage (Alcohol Use Disorder Identification Test (AUDIT) [37] and Alcohol, Smoking and Substance Involvement Screening Tool (ASSIST) [37]), mental health and well-being (Strengths and Difficulties Questionnaire (SDQ) [38, 39] and Warwick-Edinburgh Mental Well-being Scale (WEMWBS) [40]) and health-related quality of life (EQ-5D-5L [41]).

#### Follow-up

All young people enrolled into the trial study will be contacted by phone and letter/email 12 months post-baseline to complete a follow-up questionnaire. The questionnaire will be administered by the researcher who will visit the young person in their home/convenient location. As in the baseline questionnaire, most data will be self-completed on a tablet. However, the Timeline Followback substance use and self-reported occasions of ‘drunkenness’ in the last 30 days [42] will be researcher administered. The researcher will also be available to provide clarification on questions as necessary. In this feasibility trial, to minimise respondent fatigue at baseline as recommended during peer review, questions relating to use of health and social services [41] and placement stability and potentially sensitive questions on sexual behaviour (ASAI) [7, 43] and antisocial/criminal behaviour [44] will be asked at follow-up only, reducing participant burden. These questions will be piloted within the feasibility trial for value of information and acceptability to the young people. In a future definitive trial, between-group differences of these secondary outcomes will be analysed at follow-up.

### Process evaluation

The process evaluation has three aims: to examine the feasibility of implementing the intervention, to understand the mechanisms through which change occurred and to consider the role of context in shaping this change [45]. In keeping with the MRC guidelines, we will use quantitative and qualitative methods to address these aims.

#### Feasibility of intervention implementation

We aim to understand and document the key lessons learned from implementing SOLID (both the interventions and the trial processes) and to evaluate factors needed to deliver the intervention at scale. With regard to the intervention delivery, we will use Carroll et al.’s definition of fidelity, i.e. we will assess whether intervention sessions are delivered as planned in terms of content, frequency, duration and coverage [46]. We propose to assess the quality of intervention delivery (treatment fidelity) by applying a validated process rating scale (UKATT PRS) [47], developed in the UKATT trial. All sessions will be audio recorded, and a 20% random sample of SBNT and MET sessions will be analysed, ensuring we sample early, mid and late sessions of both interventions. UKATT PRS specifically covers MET and SBNT and assesses items including commitment, optimism, collaboration and interpersonal focus, which help determine if the LAC are actively engaged in the intervention sessions.

#### Mechanism of change

Interview schedules for staff (drug and alcohol workers and social workers) will be constructed to highlight the four core concepts of the Normalisation Process Theory [48]: coherence, cognitive participation, collective action and reflexive monitoring. Qualitative data will be collected through interviews and focus groups with social workers and drug and alcohol workers. Data collection will continue until saturation; however, as a minimum, we will carry out qualitative semi-structured interviews with 15 drug and alcohol workers and two focus groups, one with social workers and one with members from the drug and alcohol teams. The workers will be purposefully sampled to ensure that there are professionals interviewed from each local authority site and each intervention arm. The analysis will inform our understanding of the mechanism of change from a practitioner perspective and consider key barriers to successful delivery and integration of the interventions at the level of the system.

We will conduct a minimum of 20 qualitative 1:1 semi-structured interviews with a purposive sample of LAC (ensuring maximum diversity with regard to gender, placement type, study arm and age) and separate interviews with their carers (*n* = 20). These interviews aim to understand how the interventions have affected the LAC themselves and how they would recommend changing the interventions to make them more effective.

#### Context

The role of context will be core to our understanding of the mechanism of change at the level of the service user (LAC) and service provider (drug and alcohol workers and social workers). Specifically, we will consider barriers and facilitators to change.

### Planned analysis

#### Statistical analysis

As a pilot feasibility trial, the main analyses will be descriptive, in order to inform the design of a future definitive study. The primary outcomes are feasibility outcomes. We will report the numbers of eligible participants seen over the recruitment period and the resulting rates of recruitment, compliance with randomisation and data completion. Non-completers will be characterised. In addition, the descriptive analysis will include participant characteristics (for example age, gender, ethnicity, substance use type, mental health and well-being, sexual health).

The pilot feasibility trial will also assess performance of potential outcome measures for a definitive trial. We will ascertain data completeness of the instruments and any potential bias in the completion of follow-up data to inform the choice of instruments in a future trial. The majority of the outcome data will be presented in simple descriptive tables presenting percentages, means and standard deviations or 5-number summary (as appropriate), for each arm of the study. We will use an intention to treat analysis. This information will be used to inform the design, choice of primary outcome, necessary sample size and approach to the analysis, of the future definitive trial.

#### Health economic analysis

The initial stage of value of information (VoI) analysis will be the construction of a mathematical decision model that will synthesise the best available existing evidence from the literature and information from the pilot trial in order to provide preliminary estimates of effects, costs and cost-effectiveness. Uncertainty around estimates of effects, costs and cost-effectiveness will be accounted for by probabilistic sensitivity analysis and estimated using Monte Carlo simulation, as cost data are unlikely to be normally distributed. Statistical imprecision will be presented as confidence intervals around differences in effectiveness, costs and cost-effectiveness. Threshold sensitivity analyses will be applied to identify the range for costs and effects in which a treatment might need to exist in to be considered cost-effective with respect to standard thresholds for cost-effectiveness (e.g. £20,000 per QALY).

The VoI analysis will build upon the results of the probabilistic sensitivity analysis to estimate the expected value of sampling information (EVSI). The EVSI will quantify the value of reducing uncertainty via collection of additional data in a definitive trial. Comparing the value of additional information with the financial and opportunity cost to generate the additional information, the expected net gain (ENG) can be calculated as the difference between the EVSI and the total cost of conducting the further research. The optimal design of a definitive trial will maximise the ENG to society with regard to sample size. The optimal sample size is that which maximises the difference between EVSI and expected total cost of the research (both the direct cost of the research itself and the opportunity cost of delaying the implementation of a worthwhile intervention whilst research is ongoing). The estimated optimal sample size will be inflated according to the rate of missing data in the trial data to achieve the optimal sample size of complete data cases and to determine the final sample size for a definitive trial.

#### Qualitative analysis

Data transcription will be carried out verbatim. Transcripts will be anonymised by removing names of any individuals mentioned in the course of the interview; a participant key will be stored separately. Transcripts will be subject to thematic analysis [49]; analysis will be an iterative process, using the constant comparative method [50], derived from grounded theory. Qualitative software (NVIVO 10) will aid in the organisation of thematic codes and categories.

As described above, the qualitative process evaluation will provide rich data on the feasibility of the interventions and on the ‘mechanisms of change’ and the role of context. This will include data on fidelity (assessment of content and interaction), acceptability (LAC and practitioner perceptions about receiving and delivering the actual interventions) and the potential ‘mechanism of change’ (whether our theory of change pathways are borne out in practice) from both the perspective of the service provider and LAC (service user). These data will allow us to assess if either of the interventions is clearly more acceptable to both service users and providers, is more likely to be integrated into routine service delivery, and has greater potential to elicit behaviour change in the young person. The data collected within the qualitative interviews and focus group will be used to draw elements of the MET and SBNT interventions together into a single ‘optimised’ intervention if appropriate. This may occur if core components of each intervention are consistently reported as being acceptable to LAC and practitioners and can be combined together in a complementary way whilst retaining the essence of each approach.

Qualitative and quantitative data will be collected simultaneously, and both sources of data will be analysed separately; however, the data from different methods will be triangulated to ‘consider where findings from each method agree (convergence), offer complementary information on the same issue (complementarity) or appear to contradict each other (discrepancy or dissonance)’ [51].

## Discussion

This is the first UK-based randomised controlled trial that will assess the feasibility of delivering behaviour change interventions to decrease drug and alcohol use and support the mental health of looked after children in the UK. Previous cross-sectional research has highlighted the high prevalence rates of substance use and mental health difficulties within the LAC population. However, there is little intervention-based research outlining the most effective interventions to be used with this high-risk group of young people. It is necessary to conduct a feasibility study prior to a larger definitive randomised controlled trial where there are significant uncertainties regarding interventions and/or trial processes [52, 53].

The study will examine the feasibility of recruiting LAC (who score ≥2 on the CRAFFT) into the trial. In addition, the ability of trained and supervised drug and alcohol practitioners to deliver the randomised intervention to acceptable levels of fidelity will be assessed. The study will also examine the feasibility of the research team retaining participants in the trial for the follow-up data collection at 12 months.

NICE guidelines (UK) recommend multiple sessions of motivational interviewing or family-based support or group-based behavioural therapy over 1 to 2 years to reduce substance use in high-risk young people [17]. Due to the complexity and length of these interventions, they are not feasible to be delivered at scale. The current study will adapt and evaluate the pilot feasibility of two alternative evidence-based behaviour change interventions: Motivational Enhancement Therapy (MET)—a concentrated form of motivational interviewing [29]; Social Behaviour and Network Therapy (SBNT)—an approach drawing from family and social interventions in substance use [54]. Both interventions involve counselling but focus on different behaviour change pathways, with internal thoughts and views shaping the decisional balance in MET compared with external, social influences in SBNT. Whilst both of these interventions have been shown to be effective at reducing substance use, less is known about their effect with young people, particularly those who are looked after by the local authority and likely to have more fragmented family relationships.

For many LAC, it is the experience of abuse or neglect from within their families that has led them to local authority care, and this may be related to the high rates of psychiatric morbidity in this population [25]. The absence of a supportive family unit has been associated with increased rates of substance use in young people [55]. SBNT has been designed to mobilise and develop support networks which are wider than just biological family and include peers; it has been found to be effective in reducing substance use in adults when delivered through routine services. Whilst interventions based on SBNT have the potential to address this central vulnerability within a LAC population, the ability to identify a suitable social support network for a LAC is undetermined. Further, the acceptability and feasibility of both MET and SBNT must be examined.

The views of social workers, drug and alcohol practitioners and LAC will be essential to understanding the acceptability of a randomised trial of behaviour change interventions for substance using LAC. These data will also inform how best to identify and intervene to reduce substance use in this vulnerable population. Thus, we will assess whether attitudinal support and appropriate processes are evident for a future definitive trial. If feasibility and acceptability is shown, the findings of this trial will be used to inform a protocol for a definitive randomised controlled trial of behaviour change interventions to reduce risky substance use in young people aged 12–20 years who are looked after by the local authority.

### Trial status

The trial commenced recruitment in November 2016 and is currently recruiting.

## Abbreviations

CMO: Chief Medical Officer
ENG: Expected net gain
EVSI: Expected value of sampling information
LAC: Looked after children
MET: Motivational Enhancement Therapy
NEET: Not in education, employment or training
NICE: National Institute for Clinical Excellence
NPS: Novel psychoactive substances
PR: Parental responsibility
QALY: Quality-adjusted life years
RCT: Randomised control trial
SBNT: Social Behaviour and Network Therapy
TLFB: Timeline followback
VoI: Value of information
